# Improvements in patient-reported outcomes with apremilast, an oral phosphodiesterase 4 inhibitor, in the treatment of moderate to severe psoriasis: results from a phase IIb randomized, controlled study

**DOI:** 10.1186/1477-7525-11-82

**Published:** 2013-05-10

**Authors:** Vibeke Strand, David Fiorentino, ChiaChi Hu, Robert M Day, Randall M Stevens, Kim A Papp

**Affiliations:** 1 Division of Immunology and Rheumatology, Stanford University, Palo Alto, California, USA; 2 Department of Dermatology, Stanford University School of Medicine, Redwood City, California, USA; 3 Celgene Corporation, Summit, New Jersey, USA; 4 Probity Medical Research, Waterloo, Ontario, Canada

**Keywords:** Apremilast, Dermatology Life Quality Index, Phosphodiesterase 4, Psoriasis, Quality of life, SF-36

## Abstract

**Background:**

Apremilast, a specific inhibitor of phosphodiesterase 4, modulates pro-inflammatory and anti-inflammatory cytokine production.

**Objectives:**

Apremilast’s effect on patient-reported outcomes (PROs) in patients with moderate to severe psoriasis was evaluated in a phase IIb randomized, controlled trial (NCT00773734).

**Methods:**

In this 16-week, placebo-controlled study, 352 patients with moderate to severe plaque psoriasis received placebo or apremilast (10, 20, or 30 mg BID). PROs included Dermatology Life Quality Index (DLQI), pruritus visual analog scale (VAS), and Short-Form Health Survey (SF-36) to assess health-related quality of life (HRQOL). Changes from baseline and patients reporting improvements ≥minimum clinically important differences (MCID) were analyzed. Correlations between changes across various PRO instruments were explored.

**Results:**

Baseline DLQI (>10 points) and SF-36 MCS and domain scores indicated impairments in HRQOL. At 16 weeks, greater improvements from baseline in DLQI scores were reported with apremilast 20 (−5.9) and 30 mg BID (−4.4) compared with placebo (1.9; *P*≤0.005 for both), and a greater proportion of patients reported improvements ≥MCID (20 mg BID, 49.4%, 30 mg BID, 44.3%) versus placebo (25.0%; *P*<0.04). Greater improvements from baseline in pruritus VAS scores were reported with apremilast 20 (−35.5%) and 30 mg BID (−43.7%) versus placebo (−6.1%; *P*≤0.005). Significant and clinically meaningful improvements in SF-36 mental component summary scores (*P*≤0.008) and Bodily Pain, Mental Health, and Role-Emotional domains were reported with all apremilast doses (*P*<0.05), and Social Functioning with 20 and 30 mg BID (*P*<0.05) and Physical Functioning with 20 mg BID (*P*<0.03). Correlations between SF-36 scores and DLQI were moderate (r>0.30 and ≤0.60) and low between SF-36 and pruritus VAS (r≤0.30), indicating they measure different aspects of the disease.

**Conclusions:**

Apremilast treatment resulted in improved HRQOL, including DLQI and pruritus VAS over 16 weeks of treatment, in patients with moderate to severe psoriasis.

## Background

Psoriasis is a chronic inflammatory disease that affects approximately 1% to 3% of the worldwide population [1,2]. Individuals with psoriasis report impaired health-related quality of life (HRQOL), ranging from physical discomfort and limitations in activities of daily living to psychosocial problems and emotional distress [3-7]. Furthermore, the severity of psoriasis symptoms, and pruritus in particular, has been linked to the degree of HRQOL impairment [3,4].

Many therapies for psoriasis treatment improve HRQOL [8-10]. Despite this, each therapy’s benefit can be compromised by poor tolerability, adverse events, and route of administration (particularly injection/infusion reactions) [11,12]. These limitations underscore the persistent unmet need for additional treatment options for psoriasis [13]. As new therapies become available for managing psoriasis, it is important to evaluate their impact on patient-reported HRQOL.

Research over the past decade in inflammatory diseases such as psoriasis has focused upon modulation of cyclic adenosine monophosphate (cAMP), a naturally occurring intracellular secondary messenger that maintains immune homeostasis by modulating production of pro-inflammatory and anti-inflammatory cytokines [14,15]. Phosphodiesterase 4 (PDE4) is a cAMP-specific PDE and the dominant PDE in inflammatory cells. PDE4 inhibition elevates intracellular cAMP, which in turn down-regulates the inflammatory response [16-19]. Apremilast (CC-10004; Celgene Corporation, Summit, NJ, USA) is a small molecule that specifically inhibits PDE4, thereby elevating intracellular cAMP levels. Elevated intracellular cAMP reduces pro-inflammatory mediators, such as tumor necrosis factor-α, interleukin-23, and interferon-γ, and increases production of anti-inflammatory mediators, such as interleukin-10 [18]. Clinical studies have demonstrated the efficacy and tolerability of apremilast in moderate to severe psoriasis and psoriatic arthritis (PsA) [20-22].

In this phase IIb, multicenter, randomized, controlled trial (RCT), orally administered apremilast (10 mg BID, 20 mg BID, or 30 mg BID) resulted in dose-dependent efficacy for the treatment of moderate to severe plaque psoriasis [20]. A significantly greater proportion of patients receiving apremilast 20 mg (28.7%) and 30 mg BID (40.9%) achieved ≥75% mean reductions from baseline in Psoriasis Area and Severity Index (PASI-75) scores compared with placebo (*P*<0.001) after 16 weeks of treatment [20]. Reductions in baseline PASI scores were evident by week 2, with a separation across doses observed by weeks 2 to 4; improvements were maintained over 24 weeks' treatment. Significant improvements in pruritus visual analog scale (VAS), static Physician’s Global Assessment, and body surface area (BSA) scores were also reported. It is hypothesized that clinical improvements would be accompanied by improvements in patient-assessed HRQOL. This report summarizes the impact of apremilast on patient-reported outcomes (PROs) over the initial 16-week placebo-controlled treatment phase of this trial.

## Materials and methods

### Study design

This phase IIb RCT was conducted at 35 sites in the United States and Canada between September 2008 and November 2009. Methods, including enrollment, study design, and procedures, have been previously published [20]. Briefly, men and women ≥18 years of age with stable, chronic, moderate to severe plaque psoriasis (PASI ≥12 and BSA ≥10% for ≥6 months) who were candidates for phototherapy or systemic therapy were enrolled and randomized 1:1:1:1 to receive apremilast 10, 20, or 30 mg BID or placebo for 16 weeks. At week 16, placebo patients were re-randomized 1:1 to apremilast 20 or 30 mg BID until week 24 in blinded fashion; all other patients continued their assigned dose of apremilast. Throughout the trial, concomitant phototherapy and use of systemic and biologic agents were prohibited. Use of topical agents was also prohibited, with the exception of Eucerin® cream for body lesions; low-potency corticosteroids for facial, axillary, and groin psoriasis lesions; and coal tar shampoo or salicylic acid preparations for scalp lesions. All patients provided written informed consent before study-related procedures were done, and the protocol and consent were approved by institutional review boards or ethics committees at all investigational sites.

### PRO assessments

Changes from baseline to week 16 and improvements ≥minimum clinically important differences (MCID) were determined for PROs, including the Dermatology Life Quality Index (DLQI), pruritus VAS scores, and Short-Form Health Survey version 2 (SF-36) (Table 1). The DLQI is a 10-item questionnaire that assesses the impact of skin disease on HRQOL over the previous week. Scores for each item range from 0 (not at all affected) to 3 (very much affected); total scores range from 0 to 30 and those >10 represent a very large impact on HRQOL [28]. The pruritus VAS assesses severity of psoriasis-related pruritus over the past 24 hours, on a 0- to 100-mm scale (0=no itch, 100=worst itch imaginable). SF-36 is a generic HRQOL questionnaire with 36 questions combined into 8 domains, scored from 0 (worst) to 100 (best). Domain scores are combined into physical (PCS) and mental component summary (MCS) scores with normative scores of 50 and standard deviations (SDs) of 10.

**Table 1:** **Overview of HRQOL assessment instruments and MCID **[23]**-**[27]

**Instrument**	**Description**	**Scale**	**MCID**
Pruritus VAS	● Pruritus visual analog scale	0–100 mm, including “anchors”	10.0 points
		(best to worst)	
DLQI	● Dermatology Life Quality Index	0–30 points	5.0 points
	● Subject report, 10 questions addressing:	(best to worst)	
	– Symptoms		
	– Feelings		
	– Daily activities		
	– Leisure		
	– Work		
	– Personal relationships		
SF-36 domains*	● Patient report, 36 items	0–100 mm	5.0 points
	– Physical Functioning	(worst to best)	
	– Role-Physical		
	– Bodily Pain		
	– General Health		
	– Vitality		
	– Role-Emotional		
	– Social Functioning		
	– Mental Health		
SF-36 PCS and MCS scores	● Calculated based upon domain scores	Normative value: mean=50, SD=10	2.5 points

*Based on transformed scale scores.

HRQOL, health-related quality of life; MCID, minimum clinically important differences; MCS, mental component summary; PCS, physical component summary; SF-36, 36-Item Short-Form Health Survey version 2.

### End points and statistical analysis

PROs were evaluated based on the intent-to-treat population using last-observation-carried-forward (LOCF) to account for missing values. Week 16 changes from baseline in DLQI and SF-36 scores were examined using ANCOVA, with treatment as a factor and baseline as a covariate. Week 16 percent change from baseline in mean pruritus VAS scores was compared using the Wilcoxon test for each apremilast group versus placebo. If either PCS and/or MCS scores of SF-36 were statistically significant, significance was tested for the individual domain scores, without *P* value corrections for multiplicity. Mean changes from baseline in domain scores are displayed using spydergrams [29], with quantification of improvements using the health utility SF-6D measure, after the method of Ara and Brazier [30,31]. As a benchmark comparison, US normative data were calculated based on age and gender distribution of the protocol population [32]. Pearson correlations (r) were determined for mean changes from baseline between the generic SF-36 Bodily Pain (BP) and Vitality (VT) domains and MCS scores at week 16 with disease-specific DLQI and pruritus VAS scores. Correlations >0.30 to ≤0.60 were considered moderate and >0.60 high [33]. These were unplanned, post hoc analyses based on results from secondary analysis of PROs, with the *P* values presented for inferential use.

## Results

### Patients

The majority of patients (N=352) were men (62.8%), white (92.9%), and obese (mean ± SD body mass index, 31.2 ± 7.1 kg/m^2^). Patients had plaque psoriasis for a mean of 19 years and 21% had PsA (Table 2).

**Table 2:** Baseline demographic and clinical characteristics of the enrolled population

**Characteristic**	**Placebo (n=88)**	**Apremilast BID**			**Total (N=352)**
		**10 mg (n=89)**	**20 mg (n=87)**	**30 mg (n=88)**	
Age (years)	44.1 ± 13.7	44.4 ± 14.0	44.6 ± 12.6	44.1 ± 14.7	44.3 ± 13.7
Male	53 (60.2)	63 (70.8)	55 (63.2)	50 (56.8)	221 (62.8)
Race					
White	83 (94.3)	82 (92.1)	82 (94.3)	80 (90.9)	327 (92.9)
Black	1 (1.1)	2 (2.2)	1 (1.1)	2 (2.3)	6 (1.7)
Asian	4 (4.5)	3 (3.4)	2 (2.3)	4 (4.5)	13 (3.7)
Other	0 (0.0)	2 (2.2)	2 (2.3)	2 (2.3)	6 (1.7)
Height (cm)	171.2 ± 8.6	171.5 ± 10.2	171.7 ± 9.6	171.2 ± 10.7	171.5 ± 9.6
Weight (kg)	90.3 ± 21.4	95.7 ± 23.2	89.9 ± 20.2	91.2 ± 23.1	91.8 ± 22.0
BMI (kg/m^2^)	30.8 ± 6.7	32.5 ± 7.4	30.4 ± 6.2	31.1 ± 7.8	31.2 ± 7.1
Total PASI score	18.1 ± 5.7	18.1 ± 6.3	18.5 ± 7.3	19.1 ± 7.1	18.5 ± 6.6
BSA	21.0 ± 11.2	21.3 ± 11.4	20.7 ± 12.4	25.0 ± 15.4	22.0 ± 12.8
+ History PsA	17 (19.3)	20 (22.5)	16 (18.4)	21 (23.9)	74 (21.0)
Plaque psoriasis history (years)	19.6 ± 11.6	18.0 ± 12.4	19.2 ± 12.2	19.2 ± 12.0	19.0 ± 12.0
Previous systemic therapy for psoriasis	39 (44.3)	47 (52.8)	43 (49.4)	47 (53.4)	176 (50.0)

Values are mean ± SD or n (%). BMI, body mass index; BSA, body surface area; PASI, Psoriasis Area and Severity Index; PsA, psoriatic arthritis.

### PRO assessments

#### DLQI

Mean DLQI scores were similar and >10 across all treatment groups at baseline (Table 3). At week 16, mean DLQI scores were significantly lower (vs. placebo) with apremilast 20 mg BID (*P*<0.001) and 30 mg BID (*P*=0.005), but not with 10 mg BID (*P*=0.132). Twenty-two (25.0%) placebo, 30 (33.7%) apremilast 10 mg BID (*P*=0.249), 43 (49.4%) 20 mg BID (*P*=0.001 vs. placebo), and 39 (44.3%) 30 mg BID (*P*=0.011 vs. placebo) patients reported improvements ≥MCID.

**Table 3:** Summary DLQI and pruritus VAS scores at week 16

**Patient-reported outcome**	**Placebo (n=88)**	**Apremilast BID**		
		**10 mg (n=89)**	**20 mg (n=86)**	**30 mg (n=88)**
DLQI				
Baseline	10.7 ± 6.7	10.8 ± 6.3	11.6 ± 7.0	10.6 ± 6.2
Week 16	8.6 ± 7.6	7.6 ± 6.3	5.8 ± 6.0	6.0 ± 6.2
Change from baseline	−1.9 ± 5.2	−3.2 ± 6.0	−5.9 ± 6.7*	−4.4 ± 5.1*
Pruritus VAS				
Baseline	55.5 ± 25.5	54.1 ± 23.5	58.3 ± 26.7	55.3 ± 25.5
Week 16	45.6 ± 30.2	39.0 ± 27.8	35.2 ± 29.2	31.6 ± 30.0
Percent change from baseline	−6.1 ± 76.4	−10.2 ± 100.8	−35.5 ± 66.0*	−43.7 ± 46.8*

*P≤0.005 vs placebo.

Values are mean ± SD. DLQI, Dermatology Life Quality Index; VAS, visual analog scale.

#### Pruritus VAS

Baseline pruritus VAS scores were well matched across treatment groups (Table 3). Significantly greater reductions from baseline in mean pruritus scores at week 16 were reported with apremilast 20 mg BID (*P*=0.005) and 30 mg BID (*P*<0.001), but not with 10 mg BID (*P*=0.366) versus placebo. Thirty-nine (44.3%) placebo, 47 (52.8%) apremilast 10 mg BID (*P*=0.294), 53 (60.9%) 20 mg BID (*P*=0.034 vs. placebo), and 56 (63.6%) 30 mg BID patients (*P*=0.015) reported improvements ≥MCID.

#### SF-36 MCS and PCS scores

**Figure 1:**
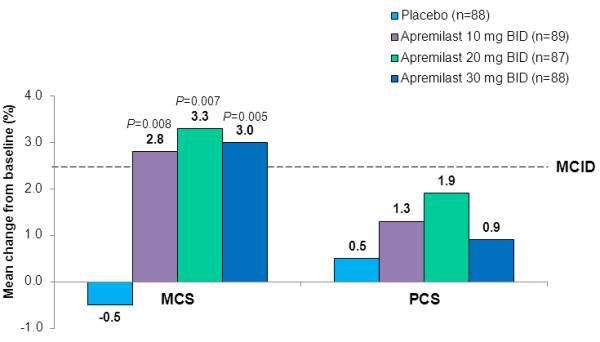
**Mean change from baseline in SF-36 physical and mental component summary scores at week 16.***P* values (vs. placebo), based on ANCOVA, with treatment as the factor, and baseline value as the covariate. An increase in score indicates improvement. MCID, minimal clinically important difference; SF-36, 36-item Short-Form Health Survey.

At baseline, mean SF-36 MCS scores across treatment groups were 0.5 SDs lower than US normative scores of 50 (range: 45.7–47.0), whereas SF-36 PCS scores approximated 50 (range 48.6–49.3). After 16 weeks of treatment, mean changes from baseline in MCS scores were significantly greater with apremilast 10, 20, or 30 mg BID than placebo (Figure 1), exceeding MCID, and approaching US normative values (range: 49.0–49.9). Week 16 mean changes from baseline in PCS scores were numerically greater with apremilast 10, 20, and 30 mg BID than placebo but were neither statistically significantly nor clinically meaningful.

#### SF-36 domain scores

At baseline, SF-36 domain scores were generally well matched between study groups and equaled or were within 10 points of age- and gender-matched normative scores. At week 16, no significant changes from baseline in any domain were evident in the placebo group.

**Figure 2:**
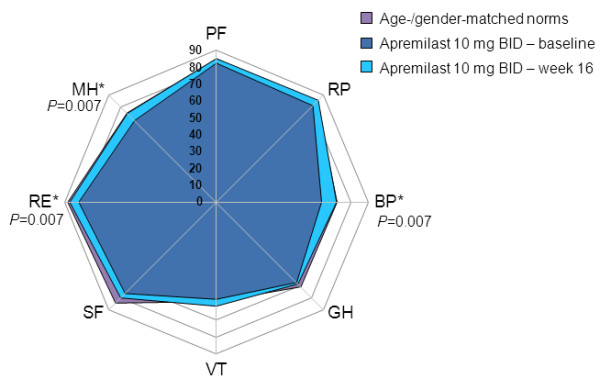
**SF-36 domain scores at baseline and week 16 with apremilast 10 mg BID.** Spydergram of SF-36 domain scores in patients receiving apremilast 10 mg BID versus US age-/gender-matched norms (lavender) and baseline (dark blue). Gridlines represent changes of 10 points each (10 points = 2× MCID). An increase in score indicates improvement. Treatment-associated improvements (light blue) are statistically significant and ≥MCID in three of eight domains. MCID, minimal clinically important difference; SF-36, 36-item Short-Form Health Survey. *≥MCID.

**Figure 3:**
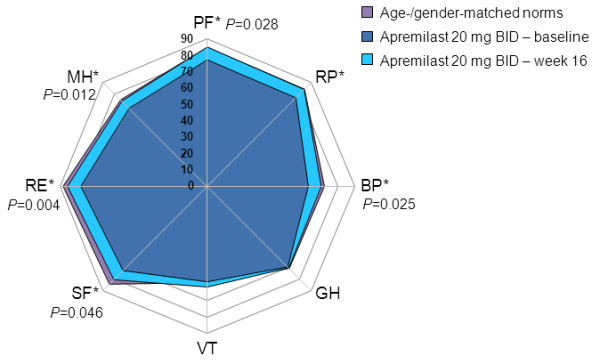
**SF-36 domain scores at baseline and week 16 with apremilast 20 mg BID.** Spydergram of SF-36 domain scores in patients receiving apremilast 20 mg BID versus US age-/gender-matched norms (lavender) and baseline (dark blue). Gridlines represent changes of 10 points each (10 points = 2× MCID). An increase in score indicates improvement. Treatment-associated improvements (light blue) are statistically significant in five and ≥MCID in six of eight domains. MCID, minimal clinically important difference; SF-36, 36-item Short-Form Health Survey. *≥MCID.

**Figure 4:**
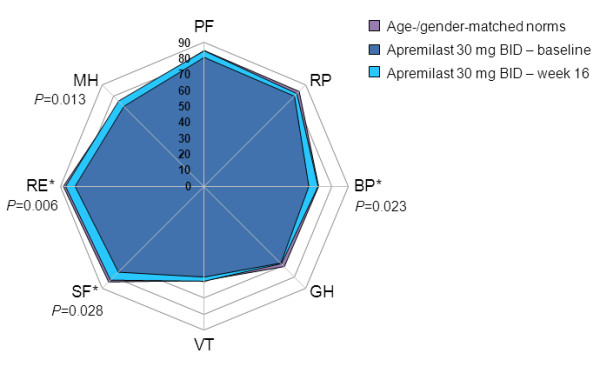
**SF-36 domain scores at baseline and week 16 with apremilast 30 mg BID.** Spydergram of SF-36 domain scores in patients receiving apremilast 30 mg BID versus US age-/gender-matched norms (lavender) and baseline (dark blue). Gridlines represent changes of 10 points each (10 = 2× MCID of 5 points). An increase in score indicates improvement. Treatment associated improvements (light blue) are statistically significant in four of eight domains and ≥MCID in three of eight domains. MCID, minimal clinically important difference; SF-36, 36-item Short-Form Health Survey.*≥MCID.

In patients receiving apremilast 10 mg BID, mean changes from baseline were statistically significant and ≥MCID in three of eight domains: BP, Mental Health (MH), and Role-Emotional (RE; *P*=0.007 for all; Figure 2). In the apremilast 20 mg BID group, mean changes from baseline were statistically significant in five domains (Physical Functioning [PF; *P*=0.028], BP [*P*=0.025], Social Functioning [SF; *P*=0.046], RE [*P*=0.004], and MH [*P*=0.012]), and ≥MCID in six domains (PF, BP, SF, RE, MH, and RP) (Figure 3). Improvements reported in the apremilast 30 mg BID group were statistically significant in four domains: BP (*P*=0.023), SF (*P*=0.028), RE (*P*=0.006), and MH (*P*=0.013) and ≥MCID in three (BP, SF, and RE) (Figure 4). Across all three active doses, statistically significant improvements were reported in BP, MH, and RE, and in SF for apremilast 20 and 30 mg BID, contributing to statistically significant changes in MCS scores. Mean changes from baseline in SF-6D scores were 0.042 and 0.052, respectively, in the apremilast 10 and 20 mg BID dose groups, exceeding minimum important differences (MID), and 0.036, approaching MID, with 30 mg BID.

#### Correlations among DLQI, pruritus VAS, and SF-36 scores

Correlations observed between generic SF-36 MCS, BP, and VT domain scores and disease-specific DLQI and pruritus VAS scores were analyzed (Table 4). With all doses of apremilast, statistically significant moderate correlations were observed between SF-36 BP domain and DLQI (*P*<0.001) and MCS scores and DLQI (*P*<0.001). Correlations between SF-36 VT domain and DLQI were overall low, but statistically significant with apremilast 20 mg (*P*=0.003) and 30 mg BID (*P*=0.040). Correlations between BP domain and pruritus VAS were moderate overall and statistically significant (*P*<0.02); correlations between VT domain and MCS scores and pruritus VAS were low, and statistically significant with apremilast 30 mg BID only.

**Table 4:** Correlations of changes in SF-36 BP, VT, and MCS scores with changes in DLQI and pruritus VAS scores at week 16

	**Placebo**	**Apremilast BID**		
		**10 mg**	**20 mg**	**30 mg**
SF-36 BP with DLQI	−0.45*	−0.32*	−0.52*	−0.35*
	*P*<0.001	*P*=0.002	*P*<0.001	*P*=0.001
SF-36 VT with DLQI	0.38*	−0.18	−0.33*	−0.23
	*P*<0.001	*P*=NS	*P*=0.003	*P*=0.040
SF-36 MCS with DLQI	−0.57*	−0.41*	−0.42*	−0.31*
	*P*<0.001	*P*<0.001	*P*<0.001	*P*=0.004
SF-36 BP with pruritus VAS	−0.39*	−0.27	−0.43*	−0.30
	*P*<0.001	*P*=0.013	*P*<0.001	*P*=0.005
SF-36 VT with pruritus VAS	−0.36*	0.01	−0.11	−0.23
	*P*=0.001	*P*=NS	*P*=NS	*P*=0.034
SF-36 MCS with pruritus VAS	−0.35*	−0.21	−0.12	−0.35*
	*P*=0.001	*P*=NS	*P*=NS	*P*=0.001

*Moderate correlations (r>0.30 and ≤0.60).

BP, Bodily Pain; DLQI, Dermatology Life Quality Index; MCS, mental component summary; SF-36, Short-Form 36 Health Survey; VAS, visual analog scale; VT, Vitality.

## Discussion

In this RCT, patients with moderate to severe plaque psoriasis reported impairments in disease-specific and generic measures of HRQOL at baseline, evidenced by mean DLQI scores >10 and SF-36 MCS scores 0.5 SDs below US age-/gender-matched norms. Statistically significant and clinically meaningful improvements in SF-36 MCS and domain scores were reported by patients treated with apremilast, most evident in the 20- and 30-mg BID dose groups, mirrored by decreases in disease-specific DLQI and pruritus VAS scores. In contrast, patients receiving placebo reported little change or deterioration from baseline in HRQOL. Correlations between SF-36 MCS, BP, and VT scores and DLQI were moderate and, in general, statistically significant. Correlations between SF-36 scores and pruritus VAS were moderate to low, indicating that they measure different impacts of the disease and highlighting the value in assessing efficacy using multiple instruments.

### Interpretation and implications

Growing evidence clearly shows that impairments in HRQOL are a large component of the disease burden imposed by psoriasis. These results are in line with national survey findings that show the majority of individuals with psoriasis experience emotional as well as physical disease-related problems [34]. In that survey, 63% of respondents reported that psoriasis impacts their emotional well-being, marked by feelings of helplessness, anger, embarrassment, and frustration. Given the generally high level of emotional distress patients report regarding psoriasis symptoms, it is important to describe the potential impact of treatment on emotional functioning. Emotional distress has been linked to onset of psoriasis flares, more severe symptoms, and presence of lesions in visible locations [3,35] and may contribute to heightened risk of major depressive disorder, often seen in this patient population [5,6,36]. This trial contributes to other RCT data demonstrating that efficacious treatment of moderate to severe psoriasis results in improvements in HRQOL [7-9,37,38]. Consistent with findings from other studies [7], psoriasis appears to have relatively greater impact on mental health rather than physical domains, as patients report larger decrements in RE and SF and to a lesser degree BP domains. This suggests that the impact of physical well-being on mental health might well depend on the nature of the physical impairment, as skin disease may have a disproportionately large effect on mental functioning and ensuing HRQOL whereas joint disease in PsA has more impact on PF, BP, and VT domains. These data demonstrate moderate correlations between improvements in disease-specific functioning, based on DLQI, and broader improvements in SF-36 MCS scores and BP, SF, and RE domains. This likely reflects improvements in painful psoriatic plaques and/or joint pain associated with comorbid PsA, although only a minority of patients reported the presence of arthritis. The ability to measure treatment efficacy based on broader generic instruments has implications when considering comparisons with normative populations as well as across disease states. Disease-associated decrements in HRQOL reported by patients with psoriasis indicate that a multipronged approach could enhance assessment of treatment effectiveness. This approach should encompass clinical signs and symptoms, as well as patient-reported HRQOL, using both disease-specific and generic instruments.

### Apremilast clinical data

As described in a separate report [20], the primary results of this study demonstrate the efficacy and tolerability of apremilast 20 and 30 mg BID over 24 weeks in patients with moderate to severe plaque psoriasis, including significant improvements in PASI, pruritus, static Physician’s Global Assessment, and BSA scores. The most frequently reported adverse events were headache, nausea, vomiting, nasopharyngitis, and upper respiratory tract infection. The majority of adverse events (>96%) were mild or moderate in severity, and rates of discontinuations due to adverse events were generally low (≈10%). Gastrointestinal events were generally transient. Importantly, no opportunistic infections were reported, and no serious infections were considered related to apremilast [20].

The tolerability findings are important in their relationship to the improved HRQOL seen in this study. While improvement in the severity of disease can be expected in most cases to improve HRQOL, there are some instances in which tolerability and safety issues may abrogate this benefit. The results of the current analysis indicate that this was not the case with apremilast. While the most commonly reported adverse events were related to gastrointestinal complaints and headache, this did not appear to outweigh the benefit of therapy, and resulted in an overall improvement in HRQOL.

On the basis of phase II findings, further examination of the efficacy and safety of apremilast for the treatment of psoriasis is underway in phase III studies. The ESTEEM (Efficacy and Safety Trial Evaluating the Effects of apreMilast in psoriasis) clinical trial program includes two 52-week RCTs, each with a 16-week placebo-controlled phase, randomized withdrawal phase for PASI responders, and long-term, open-label extension to assess the efficacy, tolerability, and effects of apremilast on HRQOL in patients with moderate to severe plaque psoriasis. Recently, topline results from the ESTEEM 1 trial were presented [39]. At week 16, a significantly greater proportion of patients receiving apremilast 30 mg BID achieved PASI-75 (33.1%) and PASI-50 (58.7%) compared with placebo (5.3% and 17.0%; *P*<0.0001 for both). Apremilast was also associated with significant improvements in static Physician’s Global Assessment, pruritus VAS, and DLQI as well as difficult-to-treat nail and scalp psoriasis. Apremilast was well-tolerated and no new safety or laboratory signals were detected. Additional results are anticipated. In addition, apremilast is currently being investigated in phase III clinical trials for the treatment of PsA and ankylosing spondylitis.

### Limitations

The study enrolled patients with moderate to severe plaque psoriasis and results may not be applicable to patients with other forms of psoriasis. This report is based on PROs after 16 weeks of treatment, although a separate publication indicates that improvements with active therapy are generally maintained over 24 weeks of treatment [20]. Ongoing phase III studies are expected to yield valuable information.

## Conclusions

Moderate to severe plaque psoriasis is associated with a negative impact on HRQOL, including pain and social and emotional functioning. Assessment of clinical signs and symptoms, as well as PROs, is useful in determining the impact of psoriasis and the clinical efficacy of treatment. In this study, apremilast 20 mg and 30 mg BID consistently resulted in statistically significant and clinically meaningful improvements in disease-specific and generic measures of HRQOL in patients with long-standing, moderate to severe plaque psoriasis. Findings indicate that the benefit:risk profile of apremilast results in a net improvement in HRQOL for the patient.

## Abbreviations

BP: Bodily Pain [cap p]; BSA: Body surface area; cAMP: Cyclic adenosine monophosphate; DLQI: Dermatology Life Quality Index; HRQOL: Health-related quality of life; LOCF: Last observation carried forward; MCID: Minimum clinically important differences; MCS: Mental component summary; PASI-75: ≥75% mean reductions from baseline in Psoriasis Area and Severity Index; PDE4: Phosphodiesterase 4; PCS: Physical component summary; PF: Physical Functioning; PROs: Patient-reported outcomes; PsA: Psoriatic arthritis; RCT: Randomized, controlled trial; RE: Role-Emotional; SDs: Standard deviations; SF: Social Functioning; SF-36: 36-item Short-Form Health Survey; VAS: Visual analog scale; VT: Vitality.

## Competing interests

VS has been a consultant to and member of scientific advisory boards for Abbott Immunology, Alder Biopharmaceuticals, Amgen, BiogenIdec, BMS, Celgene Corporation, Galderma, Idera, Incyte, Janssen, MedImmune, Novartis, Pfizer Inc, Regeneron, Rigel, Roche, Sanofi, and UCB. DF has served as an investigator for Amgen, Celgene Corporation, Janssen, Pfizer Inc, and Roche, and as a paid advisor for Amgen, Janssen, MedImmune, and Rigel. CH, RMD, and RMS are employees of Celgene Corporation. KAP has served as an investigator for Abbott, Amgen, Boehringer Ingelheim, BMS, Celgene Corporation, Centocor, Galderma, Isotechnika, Janssen, Leo Pharma, Lilly, MedImmune, Merck, Novartis, and Pfizer Inc; an adviser for Abbott, Amgen, Astellas, BMS, Celgene Corporation, Centocor, Galderma, Incyte, Isotechnika, Janssen, Johnson & Johnson, Lilly, MedImmune, Merck, Novartis, Pfizer Inc, and UCB; and a speaker for Abbott, Amgen, Astellas, Celgene Corporation, Centocor, Isotechnika, Janssen, Novartis, and Pfizer Inc.

## Authors’ contributions

All authors had full access to all of the data in the study. VS, DF, CH, RMD, RMS, KAP: Conceived of the study, participated in the design and coordination of the study, and helped to draft the manuscript. CH, RMD, RMS, KAP: Supervised on and performed data analyses and helped to draft the manuscript. VS, CH, RMD, RMS, KAP: Analyzed and interpreted the data and helped to draft the manuscript. VS, DF, CH, RMD, RMS, KAP: All authors had full access to the data and have read and approved the final manuscript.
